# Some Risk Factors of Chronic Functional Constipation Identified in a Pediatric Population Sample from Romania

**DOI:** 10.1155/2016/3989721

**Published:** 2016-11-23

**Authors:** Claudia Olaru, Smaranda Diaconescu, Laura Trandafir, Nicoleta Gimiga, Gabriela Stefanescu, Gabriela Ciubotariu, Marin Burlea

**Affiliations:** ^1^“Gr. T. Popa” University of Medicine and Pharmacy, Iași, Romania; ^2^“Sf. Maria” Emergency Hospital for Children, Iași, Romania; ^3^“Sf. Spiridon” Emergency Hospital, Iași, Romania

## Abstract

We conducted an observational study over a 1-year period, including 234 children aged 4–18 years and their caregivers and a matching control group. 60.73% of the children from the study group were males. Average age for the onset of constipation was 26.39 months. The frequency of defecation was 1/4.59 days (1/1.13 days in the control group). 38.49% of the patients in the sample group had a positive family history of functional constipation. The majority of children with functional constipation come from single-parent families, are raised by relatives, or come from orphanages. Constipated subjects had their last meal of the day at later hours and consumed fast foods more frequently than the children in the control sample. We found a statistically significant difference between groups regarding obesity/overweight and constipation (*χ* ^2^ = 104.94, df = 2, *p* < 0.001) and regarding physical activity and constipation (*χ* ^2^ = 18.419; df = 3; *p* < 0.001). There was a positive correlation between the number of hours spent watching television/using the computer and the occurrence of the disease (*F* = 92.162, *p* < 0.001, and 95% Cl). Children from broken families, with positive family history, defective dietary habits, obesity and sedentary behavior, are at higher risk to develop chronic functional constipation.

## 1. Introduction

Chronic constipation is a disorder that can negatively impact the quality of life, resulting in a major economic and social burden. Among the pediatric population, constipation is a frequent disorder with a growing incidence ranging from 3% to 25% [1]. Constipation is also a condition based on clinical symptomatology, with its definition being particularly subjective [2, 3]. In this regard, there is often a lack of understanding between the physician and the patients' perception in terms of defining constipation [4]. The Rome III criteria consider the frequency and consistency of stools, as well as the secondary disorders associated with constipation (retentive behavior, pain).

*The Definition of Functional Constipation according to the Rome III Criteria [5–7]*. In children over the age of 4, at least 2 of the following events occur, once a week or more frequently, for a duration of at least 2 months:

- Less than 3 bowel movements per week
- At least 1 episode of fecal incontinence per week
- Stool retention position or behavior
- Difficult or painful passing of stool
- The presence of a large fecal mass identified upon rectal examination or palpation of the abdomen
- The existence of large stools that can obstruct the toilet

The aim of this study is to investigate some of the possible risk factors of functional constipation and determine some correlations with diet and sociofamilial factors on a sample of children admitted into a pediatric gastroenterology unit from a tertiary hospital in Romania. The objectives of the study focused on characterizing the patient's family-related environment, analyzing hereditary-collateral antecedents, and identifying the positive history of functional constipation of the next of kin, as well as developing a nutritional profile of the patients.

## 2. Material and Method

We have conducted an observational clinical study on a sample of children with ages ranging from 4 to 18 years, diagnosed with chronic constipation defined as per the Rome III criteria. The inclusion criteria required the diagnosis of functional constipation according to the Rome III criteria, as well as obtaining the next of kin's consent for participating in the study. The exclusion criteria targeted the causes of organic constipation (Hirschsprung disease, pseudo bowel obstruction, previous surgical interventions in the anus/colon, or neurological disorders: hypotonia, cerebral palsy, and severe mental retardation). Of the total number of children admitted for constipation we excluded 48 children because they did not meet the definition criteria or had constipation due to organic causes; also, for 31 children we were unable to obtain the consent of their next of kin for participating in the study. The final sample comprised 234 children with chronic functional constipation. For the control group we have selected children with ages ranging from 4 to 18 years, who came to our unit for well-child visit. The inclusion criteria focused on the absence of prior episodes of chronic constipation and/or fecal incontinence, cerebral palsy, mental retardation, or other chronic diseases. The control group comprised 112 children. Consent was obtained from the next of kin and from children over the age of 14 before commencing the study. We collected data regarding the sociofamilial environment and family history in order to identify the disease in first-degree relatives. Both parents and school aged children were asked to respond to a questionnaire that included data regarding the onset of bowel movement disorders, quantitative and qualitative aspects of passed stools, determining when the signs of the disease had occurred and the connection with some extradigestive pathological events or the inclusion of certain foods in the child's diet, and conducting a dietary inquiry for the purpose of determining the type of diet leading to the onset of constipation and after that date. We assessed the nutritional status based on the following formula: BMI (kg/m^2^) = W (kg)/H (m^2^). Excess weight was associated with BMI over the 85th percentile of the baseline standard, obesity meant BMI over the 95th percentile, and weight hypotrophy was defined as BMI below the 5th percentile. The nutritional status of the adults was also assessed using the BMI. They were deemed underweight if BMI ≤ 18.5 kg/m^2^, normal (weight) if 18.5 < BMI < 24.9 kg/m^2^, overweight if 25.0 < BMI < 29.9 kg/m^2^, and obese if BMI ≥ 30 kg/m^2^. The adults were diagnosed with functional constipation according to the Roma III criteria for adults. We also assessed their level of physical activity by determining the number of days/week with more than 60 minutes moderate-vigorous physical activity and we quantified the time they spend watching television or using the computer. Data processing was carried out using the SPSS 20.0 software.

## 3. Results

**Figure 1:**
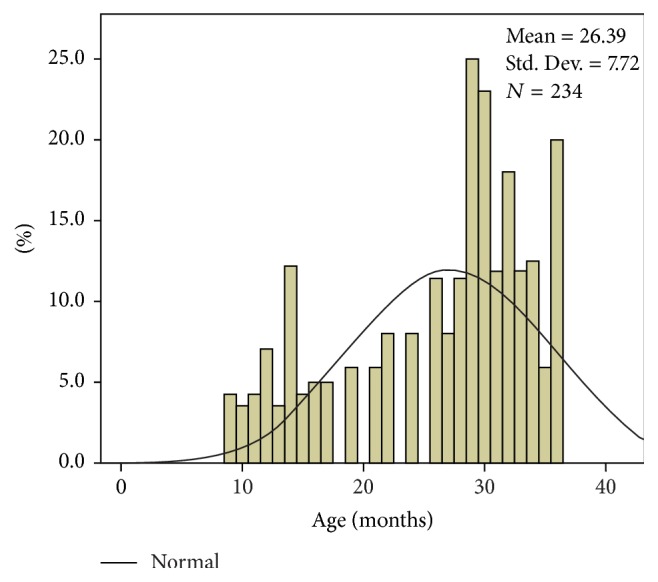
Constipation onset age distribution histogram.

142 of the 234 children that went through with the study were males (60.73%) and 92 (39.27%) were females. There were no significant gender differences in the control sample, with an M : F ratio of 1 : 1.1. As for geographical area of origin, there were no significant differences between the two samples, both showcasing a slight prevalence of patients from urban areas (53% in the patient series and 53.65% in the control group). Average age for the onset of the symptomatology was 26.39 months, with minimum values of 9 months and maximum values of 36 months. We noted that 63.14% of the cases entered in the study stated that the onset of constipation occurred around the age of 24–36 months, with a calculated average of 26.39 months (Figure 1).

**Figure 2:**
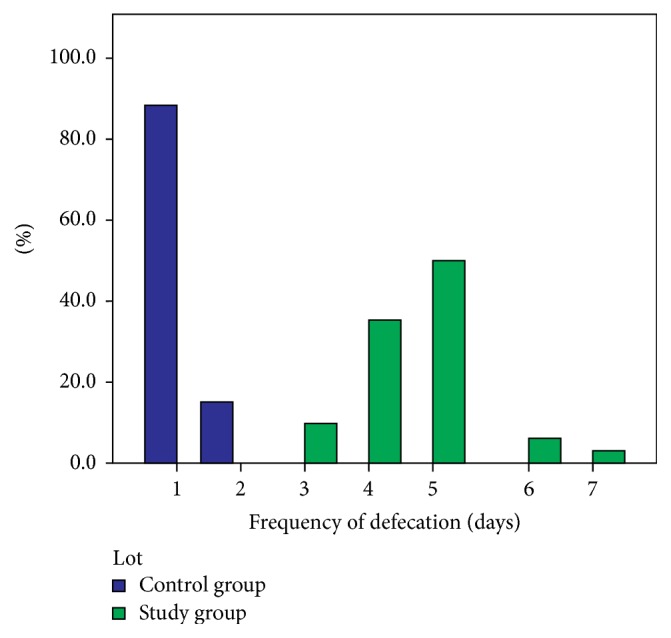
Distribution of patient samples per frequency of bowel movements.

As far as the number of bowel movements is concerned, we noticed that their frequency was significantly lower in the children in our study sample, who reported an average of 1 bowel movement per 4.59 days compared to 1 bowel movement per 1.13 days in the control sample (Figure 2).

**Figure 3:**
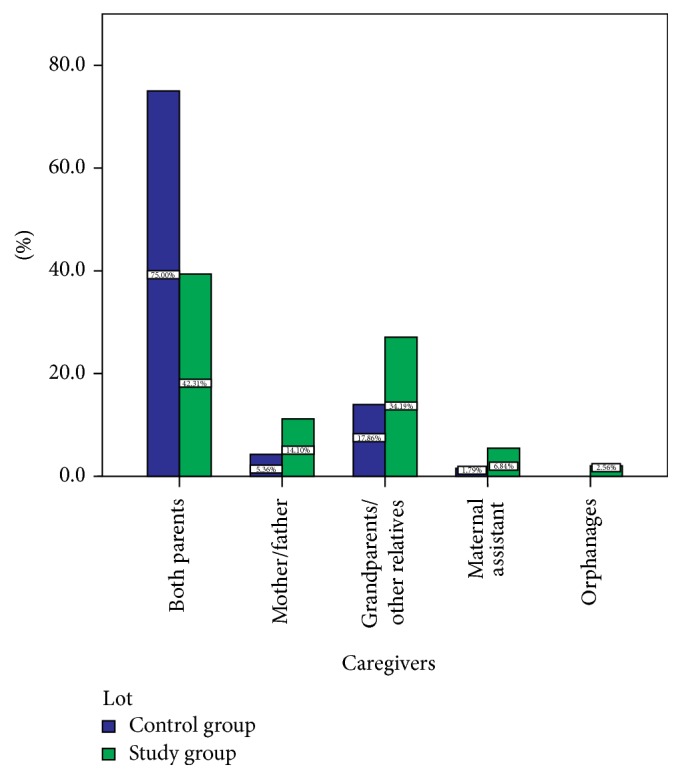
Sample distribution per sociofamilial environment.

90 of the 234 patients in the studied sample had a positive family history of functional constipation (38.49%). Of all these cases, constipation was reported in the mother in 61 of cases (46.56%) and in the father in 29 cases (22.14%), and in 20 cases constipation was identified in the siblings (15.27%). In 21 (16.03%) of these cases 2 members of the family were affected. In the control sample, only 3 parents reported this condition. As for the sociofamilial environment, 80 (34.19%) of the children did not live with their parents (they lived with their grandparents or other relatives), 33 (14.1%) lived in single-parent households, 16 (6.84%) lived with maternal assistant, and 6 (2.56%) came from orphanages. A significant aspect was that only 99 (42.31%) of the patients lived with both of their parents compared to 75% of the children in the control sample (Figure 3).

**Figure 4:**
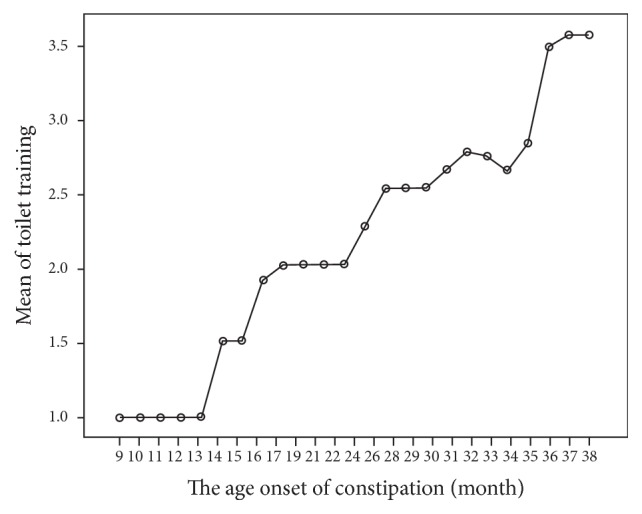
Correlation between the age of onset of constipation and the moment when toilet training was initiated.

The age for starting toilet training in the studied sample ranged from 1 year to 3.5 years, with an average of 2.4 years compared to 1.8 years in the control sample. The average age for initiating toilet training was significantly higher among the children in the patient sample as compared to those in the control sample (*F* = 58.744, *p* < 0.001, and 95% Cl). There is a statistical correlation between the average age for initiating toilet training and the age of onset of constipation (*F* = 70.749, *p* < 0.001, and 95% Cl) ((Table 1) and (Figure 4)).

The dietary inquiry for the first year of life showed that a low percentage of the children in the patient sample were breastfed, namely, 26.07% (60.71% in the control sample), while 40.60% were fed with cow's milk (with the other patients being fed with formula or mixed feeding). In the control sample the ratio of cow's milk based diet was of just 7.14%. The statistical analysis of cases in both of the studied samples based on the type of milk in their diet shows a significant statistical difference (*χ* ^2^ = 55.60; df = 3; *p* < 0.00); therefore, we can presume that cow's milk based diets for the first year of life favor the occurrence of constipation. The patients' original environment influenced their diet during the first year of life; thus, a higher frequency of children fed with cow's milk was identified in those living in rural areas (78.95%). 78.26% of the patients fed with formula were from urban areas. Food diversification was carried out incorrectly in 21.82% (51) of the patients compared to 19.61% (22) of the children in the control sample, starting at the age of 3 months. Average age for beginning food diversification was 4.22 months. As for their current nutritional status and dietary habits, we identified the following aspects: of the children with chronic constipation, 69 patients were overweight (29.49%) compared to 10 (8.93%) in the control sample; 19 children (8.12%) were underweight compared to 12 (10.71%). The weight of all the other patients ranged within normal limits. We could thus note a higher prevalence of obesity in the children in the studied sample compared to the control sample. The statistical analysis revealed a statistically significant difference regarding the nutritional status of children affected by constipation (*χ* ^2^ = 104.94, df = 2, and *p* < 0.001), with obesity constituting a risk factor in the occurrence of bowel movement disorders. Then we assessed the nutritional status of all the patients' next of kin. 60.68% of the parents of the children in the studied group were overweight or obese compared to 26.79% in the control sample. In addition to this, 69.57% of the overweight/obese children in the studied group also had overweight/obese next of kin. As for the children with normal weight, only 56.85% of them had overweight/obese next of kin. The analysis of the relationship between the nutritional status of the children and their next of kin shows a significant difference (*χ* ^2^ = 63.49; df = 4; *p* < 0.001); therefore, we could state that overweight/obese parents have a greater likelihood to have an overweight/obese child. The nutritional inquiry tracked the number and schedule of daily meals (Table 2). We also investigated the fast food intake and the type of foods consumed.

**Figure 5:**
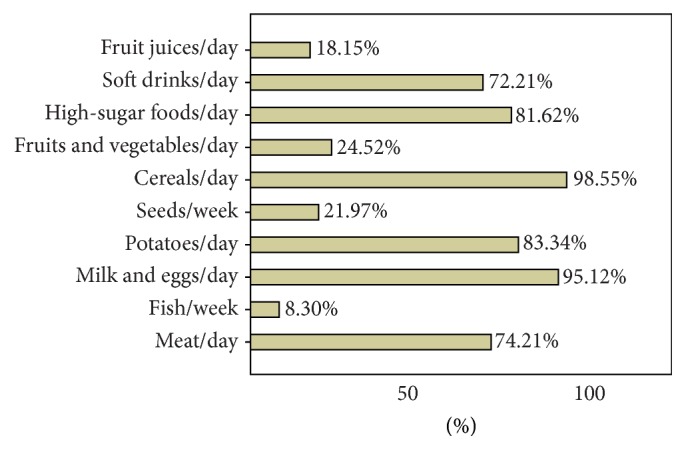
Nutritional profile of the child with constipation.

We noted that 24.41% of the children in the studied sample ate 3 times a day, 58.13% stated they had 4 meals/day, and 17.46% reported 5 meals/day (3 main meals and 2 snacks). The calculated average was 3.93 meals/day. A significant percentage of the control sample patients had 3 main meals/day (73.22%), and only 6.23% reported eating 5 meals (3 main meals and 2 snacks). In order to identify erratic meal schedules, we investigated the time of the last meal. The reported times ranged from 19:00 to 23:00, with an average of 21:16 in the studied sample compared to 19:50 in the control sample. Thus, the children with functional constipation had their last meals at later hours than those in the control sample. To identify the “inadequate diet” risk factor, we considered fast foods in particular, as they were found both in the children in the studied sample and in those in the control sample. However, there is a noticeable difference in terms of frequency of consumption. Thus, the monthly average in the studied sample was 3.65 meals/month compared to the monthly average of 1.36 fast food type meals/moth in the control group. The statistical analysis indicates a correlation between the geographical area of origin and the number of fast food meals/month (*χ* ^2^ = 28,760; df = 01; *p* = 0.001). We wanted to identify a nutritional profile of the patient with constipation. For this purpose, we analyzed the frequency of consumption for the main food groups. Constipated subjects consumed the following foods more frequently: meat products, milk, concentrated sweets, and soft drinks (Figure 5). The subjects in the control sample consumed all of these products less frequently, also noting a larger intake of vegetables and fruits.

**Figure 6:**
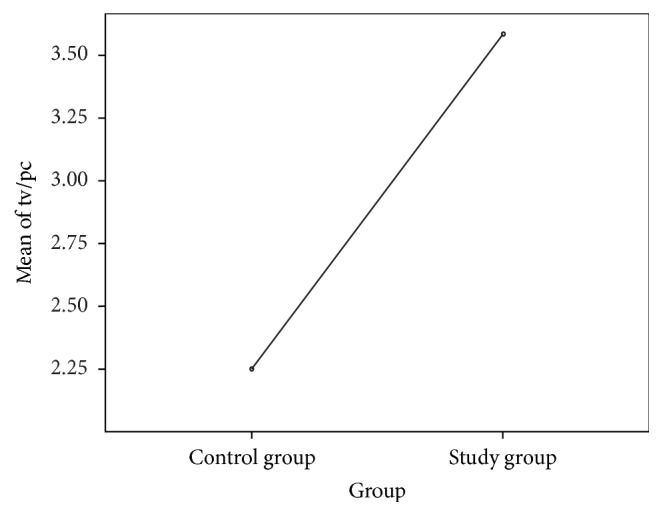
Comparison of average number of hours spent watching television/using the computer within the two studied samples.

A difference between the two samples was noticed in terms of the quantity of milk consumed on a daily basis. Among children with constipation, this quantity ranged from 200 mL to 1 liter, while in the control sample milk consumption did not exceed 500 mL/day. The average consumption in the studied sample was 563.25 mL compared to 365.63 mL in the control sample. The statistical analysis of the cases studied based on cow's milk consumption in the two samples revealed a significant difference between expected counts and actual counts (*χ* ^2^ = 210.284; df = 11; *p* < 0.001). After the interpretation of adjusted residuals we found that the number of children consuming over 600 mL of cow's milk per day is statistically significantly higher in the patient sample compared to those in the control sample. As far as physical activity is concerned, 52.99% of the children in the patient sample were not involved in any physical activity and 39.32% reported only 2 days/week of 60-minute moderate-vigorous physical activity at the most. In the control sample 33.93% had no physical activity and 59.82% reported 2–4 days/week of 60-minute moderate-vigorous physical activity. The statistical analysis of the cases studied based on the level of physical activity in the two samples revealed a significant difference (*χ* ^2^ = 18.419; df = 3; *p* < 0.001), which emphasizes the fact that lack of exercise constitutes a risk factor in the occurrence of constipation. The patients in the studied sample reported longer periods of time spent watching television or using the computer. 50% of the children in the patient sample spend around four to six hours a day watching television or using the computer, as compared to the control sample where the recorded percentage was 15.18%. Most of the children in the control sample (84.82%) spent around one and three hours/day doing these activities, as compared to the 50% of the children in the patient sample. We have thus determined a significant statistical correlation between the number of hours spent watching television/using the computer and the occurrence of constipation (*F* = 92.162, *p* < 0.001, and 95% Cl) ((Table 3) and (Figure 6)).

## 4. Discussions

In the studied sample we noticed a higher prevalence of males. These results are not consistent with other studies regarding functional constipation during childhood, which report that this disorder has a similar prevalence in boys and girls [8]. We also identified a larger ratio of patients from urban areas. This finding was interpreted in the context of improved access to medical services for patients living in urban areas. There is little epidemiological data available regarding the age of onset of constipation in infants [9]. It was reported that the onset of constipation can occur during the first year of life in approximately 50% of the affected children [10]. On the other hand, Loening-Baucke assesses the prevalence rates for constipation during the first and second year of life at 2.9 and 10.1%, respectively [11]. Our results indicate that the majority of constipated children included in this study had the onset of constipation within the first two years of life; these results are consistent with other previous studies [12–14]. The number of doctor visits for children aged 0 to 9 years on account of constipation problems has doubled in recent years, and the highest increase was reported among children under the age of 2 years [15, 16]. It is not clear whether this increase is due to an increase in the frequency of constipation episodes or to an increased number of parents requiring medical assistance for their children [17]. In our study, children affected by constipation had an average frequency of bowel movements of 1-time defecation per 4.59 days compared to 1-time defecation per 1.13 days in the control sample. Our findings are consistent with other studies; Nyhan et al. conducted a study on a sample of 800 children and reported a frequency of 1 bowel movement per 4.4 days, with the type of diet (breastfeeding or formula) also having an impact on this difference [18, 19]. Another risk factor seems to be a positive family history of functional constipation, which was reported in 38.49% of the cases in our study. The high frequency of constipation noted in the relatives of the patient series can be a result of both genetic factors and of specific eating habits that are common to all the members of the family, which might suggest that the diet followed within the household could trigger the onset of the symptomatology [2, 3, 20]. Family environment is particularly important. Although nowadays there is an ever-increasing number of grandparents acting as surrogate parents for their grandchildren, there is little data available on how these children are affected [21]. It was determined that psychological stress in the grandparents leads them to become parents of a lower quality, which ultimately leads to a higher level of maladjustment in their grandchildren [22]. Psychological stress and the feeling of insecurity—aspects that are frequently identified in children from single-parent households or institutionalized children—may have an impact on certain pathologic conditions in children [23]. Our findings show that the majority of children with functional constipation come from single-parent families, are raised by relatives, or come from orphanages. Toilet training could be yet another important factor involved in the etiology of functional constipation in children. An increase of the age for starting toilet training has been noted in several countries [24, 25]. In one of their studies, Largo et al. reported that 97% of children gained complete bowel control by the age of 3 years, these results being similar to our study where daytime bowel control was gained at the age of 2.4 years [26]. Toilet training is initiated quite late in children, most of the times after the age of 2 years, which could be explained by the involvement of both parents in their professional and social life and the convenient use of single use diapers, as well as the sheer neglect of toilet training in children from single-parent households or institutionalized ones. Only 26.07% of the children in the patient sample were breastfed, a rather small percentage compared to the control sample (60.71%). As discussed by others breastfed infants can have greater variability than formula-fed infants in stool frequency [27]. Cow's milk based diets play an important part in delaying bowel movement in infants and small children. In our study, the consumption of cow's milk was reported in 40.60% of the children with constipation (7.14% in the control sample). Unfortunately, the cow's milk based diet during the first year of life is a relatively frequent practice in poor rural communities throughout Romania. Most of the studies in the literature refer to the relationship between constipation and cow's milk protein allergy, and it was recently agreed that constipation could be the result of hypersensitivity to cow's milk proteins [28–30]. Although not clinically proved, excessive consumption of cow's milk could contribute to the occurrence of constipation even though the children are not intolerant to cow's milk proteins. Cow's milk determines slowing down of intestinal motility and triggers the satiety of the child, thus reducing the intake of fluids and other foods that contain fibers. This mechanism can be most relevant for small children, who are most susceptible to having a high intake of milk and a low intake of dietary fibers; therefore, it is recommended that cow's milk intake should be limited to 24 ounces (720 mL) per day [31, 32]. The infants and small children with chronic constipation in the studied sample had consumed a larger quantity of cow's milk (563.25 mL/day) than the children in the control sample (365.63 mL/day). An association between obesity/overweight and functional constipation in children has been reported by several other authors [33]. In our study sample, 69 (29.49%) of the patients were overweight and so were 60.68% of their parents. In the control sample, obesity/overweight could only be identified in 10 (8.93%) of the children and 26.79% of their next of kin. Previous studies report that the higher prevalence of obesity may be a result of dietary factors, activity level, or hormonal influences [33]. This determined us to investigate the existence of defective dietary habits, given the fact that nutritional behavior of children is strongly influenced by their family environment. The dietary environment within the family includes the parents' own dietary behaviors and the child feeding practices. Parents shape the development of dietary habits in children, not only in terms of foods, but also in terms of their own eating style and meal schedule [34, 35]. Maternal dietary habits are associated with the dietary habits of children, including the specific style of eating, choosing foods, and food preferences, as well as the regulation of energy intake [36, 37]. The children with constipation included in our study had their last meal of the day at later hours and consumed fast foods more frequently than the children in the control sample. The analysis of the main food groups revealed that constipated subjects consumed more frequently the following: meat products, milk, concentrated sweets, soft beverages, and less fruits and vegetables. Our findings are consistent with other reports regarding dietary recommendations in constipated children [27]. This study showed that sedentary behavior was more prevalent in constipated children. The majority of patients in the sample have limited or no physical activity. Our results are different from a study conducted on teenagers in Taiwan, but they do agree with another study conducted on preschoolers whose physical activity of less than an hour per day was associated with constipation [38, 39]. The possible mechanisms that enable physical activity to influence the frequency of bowel movements include the reduction of colonic transit time and the hormonal changes that occur during exercise [40]. Our research has some limitations. This is a single center report that only included children admitted to our gastroenterology unit. The lack of sanitary education and the insufficient dissemination of the functional constipation criteria towards and from family doctors limit referral to specialized centers only to children with evident symptoms (for instance, less than 3 bowel movements/week or painful passing of stool); we believe that the prevalence of this disorder among the children in our country might be higher; further population-based studies are necessary. In the same time, considering the reports of the World Health Organization, Romanian Health Ministry, and other studies regarding defective dietary habits, obesity, and sedentary behavior of children in our country, their association with functional constipation requires nationwide investigation [41, 42].

## 5. Conclusions

Our study reveals the association of functional constipation with male gender, in children originating from urban areas and broken homes and having defective dietary habits as suggested by a higher prevalence of obesity/overweight both in patients and in their next of kin. The dietary habits during the first year of life, as well as subsequent years—particularly the type of foods—also plays an important role and so does the children's lifestyle in terms of physical activity and using the computer. For raising awareness among both parents and children regarding these risk factors, adequate sanitary education programs in our country should be carried.

## Figures and Tables

**Table 1:** ANOVA comparison test for the age of onset of constipation and the time when toilet training was initiated.

	Sum of squares	Df	Mean square	*F*	Sig.
Between groups	111.845	23	4.863	70.749	0.000
Within groups	14.434	210	.069		
Total	126.279	233			

**Table 2:** The number of meals/day between groups.

Meals/day	Frequency	Percent
Study group		
3	57	24.41
4	136	58.13
5	41	17.46
Total	234	100.0
Control group		
3	82	73.22
4	23	20.55
5	7	6.23
Total	112	100.0

**Table 3:** ANOVA comparison test for the average number of hours spent watching television/using the computer within the two studied samples.

TV/PC	Sum of squares	Df	Mean square	*F*	Sig.
Between groups	130.499	1	130.499	92.162	0.000
Within groups	487.096	344	1.416		
Total	617.595	345			
